# Phase 2 PIONEER Trial of Oral T3D‐959 for the Treatment of Patients Diagnosed with Mild‐to‐Moderate Alzheimer’s Disease: New Results in a Modified Intent‐to‐Treat Population

**DOI:** 10.1002/alz.095625

**Published:** 2025-01-09

**Authors:** Jessica Stanek, Blake Swearingen, Warren Strittmatter, Stan Chamberlain, Hoda Gabriel, Christopher Coutlee, Charles Lineberry, John Didsbury

**Affiliations:** ^1^ T3D Therapeutics, Inc., Research Triangle Park, NC USA

## Abstract

**Background:**

PIONEER was a Phase 2 randomized, placebo‐controlled, multi‐center trial evaluating efficacy and safety of the dual PPARδ/γ agonist T3D‐959 in patients with mild‐to‐moderate Alzheimer’s disease (AD).

**Method:**

Enrolled patients (N = 250) had mild‐to‐moderate AD per NIA‐AA criteria. No AD biomarker enrollment criteria were defined. Study data revealed a cluster of sites with common data irregularities, including (1) no drug in plasma at end of treatment in patients assigned to drug (2) baseline p‐tau217 ratio inconsistent with biological AD, and (3) placebo response outside of historic norms for mild‐to‐moderate AD. These sites were excluded to form an mITT study population. Primary endpoints were ADAS‐Cog11 and ADCS‐CGIC. Secondary endpoints were plasma Aβ42/40 ratio and Digit Symbol Coding Test. Proteomic biomarkers including p‐tau217 and neurogranin were exploratory endpoints. Efficacy was evaluated as least‐squares mean changes from baseline to week 24.

**Result:**

141 patients were included in the mITT population randomized to placebo (n = 32) or T3D‐959 (15mg, n = 38; 30mg, n = 39; or 45mg, n = 32). Both primary endpoints were met, approaching statistical significance, ADAS‐Cog11 in the 30mg group (0.73 vs 2.70; p = 0.073) and ADCS‐CGIC in the 15mg group (0.39 vs 0.86; *P* = 0.060), consistent with slowing of clinical decline. Secondary endpoint plasma Aβ42/40 ratio was met, with significant improvement in the T3D‐959 30mg and 45mg groups vs placebo (30mg: *P* = 0.011; 45mg: *P* = 0.033), with a similar magnitude of effect as lecanemab at 6‐months. T3D‐959 30mg and 45mg groups significantly improved on neurodegeneration biomarker neurogranin (T3D‐959 30mg: *P* = 0.035; T3D‐959 45mg: *P* = 0.051) and changed proteomic markers suggesting pluripotent effects on dysregulated AD pathways including inflammation, oxidative stress and metabolism. T3D‐959 was safe and well‐tolerated.

**Conclusion:**

Co‐primary endpoints ADAS‐Cog11 and ADCS‐CGIC were met per protocol, approaching statistical significance at week 24. T3D‐959 demonstrated significant improvement over placebo in plasma Aβ42/40 ratio and in plasma neurogranin. Biomarkers of all three AD diagnostic criteria, Amyloid/Tau/Neurodegeneration, were improved, as well as markers of inflammation, insulin resistance and dysfunctional lipid metabolism. Results demonstrate a strong safety profile and evidence of slowing of clinical and biological decline.

